# Incidence of venous thromboembolism in Korea from 2009 to 2013

**DOI:** 10.1371/journal.pone.0191897

**Published:** 2018-01-25

**Authors:** Junshik Hong, Ju Hyun Lee, Ho-Young Yhim, Won-Il Choi, Soo-Mee Bang, Heeyoung Lee, Doyeun Oh

**Affiliations:** 1 Department of Internal Medicine, Seoul National University College of Medicine, Seoul National University Hospital, Seoul, Korea; 2 Department of Internal Medicine, Seoul National University College of Medicine, Seoul National University Bundang Hospital, Seongnam, Korea; 3 Department of Internal Medicine, Chonbuk National University Medical School, Jeonju, Korea; 4 Department of Internal Medicine, Keimyung University School of Medicine, Keimyung University Dungan Medical Center, Daegu, Korea; 5 Center for Preventive Medicine and Pubic Health, Seoul National University Bundang Hospital, Seongnam, Korea; 6 Department of Internal Medicine, School of Medicine, CHA university, CHA Bundang Medical Center, Seongnam, Korea; Universite de Bretagne Occidentale, FRANCE

## Abstract

The incidence of venous thromboembolism (VTE) is lower in Asian populations than in Western populations. The objective of the present study was to evaluate the annual age- and sex-adjusted incidence (ASR) of VTE from 2009 to 2013 in South Korea. In addition, annual change in the pattern of VTE treatment during the study period was estimated because a new direct oral anticoagulant (DOAC) had become available and was covered by health insurance in Korea beginning in January 2013. VTE cases from 2009 to 2013 were retrospectively identified based on both diagnostic and medication codes of anticoagulants used for initial treatment using the Korean Health Insurance Review and Assessment Service (HIRA) databases. The incidence of VTE increased yearly. It was significantly higher in the older population than in the younger population, and it was higher in females than in males. In 2009, ASRs of VTE, deep vein thrombosis, and pulmonary embolism were 21.3, 8.1, and 13.2 cases per 100,000 individuals, respectively in 2009. These increased to 29.2, 12.7, and 16.6 cases per 100,000, respectively, in 2013. Prescription rates of warfarin and low-molecular–weight heparin decreased with the introduction of a new anticoagulant in 2013. The proportion of subjects who underwent mechanical procedures decreased annually. The ASR of VTE in Korea continuously increased from 2009 to 2013, reflecting an increased awareness and detection of VTE as well as improved survival of patients with cancer and other morbidities. Following its introduction, DOAC rapidly replaced other anticoagulants for the treatment of VTE.

## Introduction

Deep vein thrombosis (DVT) of the lower extremity and pulmonary embolism (PE) are the most common and potentially fatal presentations of venous thrombosis. Venous thromboembolism (VTE), including DVT of the lower extremity and PE, is associated with reduced survival [1] and considerable economic burden [2]. In western countries, the annual incidence of VTE ranges from 114 to 184 cases per 100,000 individuals [1, 3–7]. Although some patients with VTE have a genetic predisposition to hypercoagulability, acquired risk factors, including immobility, cancer, surgery, pregnancy, and estrogen therapy, can promote VTE. Older people are at increased risk of VTE because most acquired risk factors are more common in older individuals and constitute multifactorial causes of VTE [8]. The incidence of VTE in Asian populations is significantly lower than in Western populations [9, 10]. However, studies from Asia since 2000 have indicated that the incidence of VTE is as high as that in Western populations under certain circumstances, including surgery [11, 12] and hospital admission [13, 14], suggesting that VTE has become a disease of major concern in Asian populations [15]. Therefore, data regarding the incidence of VTE and the annual change in incidence are crucial to understanding the effects of VTE on social and medical aspects of Asian population at present and in the future.

In 2011, a nationwide population-based epidemiologic study of annual age- and sex-standardized incidence (ASR) of VTE from 2004 to 2008 using the Korean Health Insurance Review and Assessment Service (HIRA) databases [17] showed a considerable increase in ASR of VTE over the study period [16]. The Korean National Health Insurance (NHI) service is operated by the Ministry of Health and Welfare of Korea and covers essentially the entire population of Korea, making this one of the largest nationwide epidemiologic studies performed in Asia at that time.

To update the data on VTE incidence in the Korean population, here we report results of an epidemiologic study of the ASR of VTE from 2009 to 2013 in South Korea. We also evaluated annual changes in the pattern of VTE treatment over the study period to take account of the fact that rivaroxaban is the first direct oral anticoagulant (DOAC) to be covered by Korean NHI for treatment of DVT or PE since January 2013.

## Materials and methods

### Data acquisition

South Korea has a compulsory public health insurance coverage system, NHI, which covers approximately 97% of the population [17]. HIRA is a government-operated organization that builds accurate review and quality assessment systems for claims for NHI. Claims data that healthcare service providers submit to HIRA for reimbursement for a service provided to patients are collected by HIRA. Because claims data for the remaining 3% of the population, who are covered by the National Medical Aid program, are also reviewed by HIRA, the HIRA database contains almost all inpatient and outpatient data from hospitals and community clinics in Korea, respectively, making a nationwide population study feasible. The number of people registered in the Korean NHI was 52,272,755 in 2016 [17]. HIRA claims data include patient age, sex, diagnostic codes, and treatment information, including surgical history, prescribed drugs with dose and duration, and procedures. More detailed information regarding the HIRA database is explained in our previous studies [16, 18]. Access to HIRA data is regulated by the Rules for Data Exploration and Utilization of the HIRA, and we used data with the approval of the HIRA data access committee. All data were delivered anonymously, and no researchers had access to any potentially identifying personal information, including names, addresses, and date of birth.

### Definition of VTE

A case was defined as VTE if both diagnostic and medication codes were identified simultaneously in a patient. In the current study, the term ‘VTE’ was limited to subjects with the following: 1) DVT of the lower extremity with diagnostic code I80.2 (DVT) or I80.3 (embolism or thrombosis of lower extremity), and 2) PE with diagnostic code I26 (pulmonary thromboembolism), I26.0 (pulmonary embolism with mention of acute cor pulmonale), or I26.9 (pulmonary embolism without mention of acute cor pulmonale). If a patient had both DVT of the lower extremity and PE, the patient was counted as PE. Therefore, the term ‘DVT’ denotes ‘DVT of the lower extremity without PE’, ‘PE’ means ‘PE with or without DVT’, and VTE is the combination of DVT and PE, henceforth in this manuscript. Subjects with venous thrombosis of other sites, such as upper extremity, intra-abdominal, or those with a venous thrombosis no specified sites (TNSS) were excluded. Concurrent medication codes for unfractionated heparin (UFH), low-molecular-weight heparins (LMWH), or DOAC (*i*.*e*., rivaroxaban) were mandatory to verify accurate detection of VTE.

Diagnostic codes were assigned by physicians, and most codes were assigned close to the date of diagnostic imaging positive for VTE in both inpatients and outpatients. However, the exact dates and results of image analysis are not available in the claims database. Among patients admitted to hospitals, the date of assignment of the first diagnostic code is the date when a physician made a diagnosis of VTE. Among outpatients, diagnostic codes for VTE were assigned at the time of the first outpatient visit with a diagnosis of VTE by physicians. Assignment of a diagnostic code for VTE was mandatory to prescribe anticoagulants. Therefore, there would be only a small time-lag between the development of symptoms and signs of VTE and assignment of the diagnostic code. There might be some delay between the date of VTE diagnosis (the date of first assignment of a VTE diagnostic code) and the date of anticoagulant initiation (the date of first assignment of a medication code for anticoagulants). However, anticoagulants were prescribed simultaneously or a short time after the diagnosis in most cases, with the exception of rare cases of contraindication to anticoagulant therapy.

### Case identification

**Fig 1:**
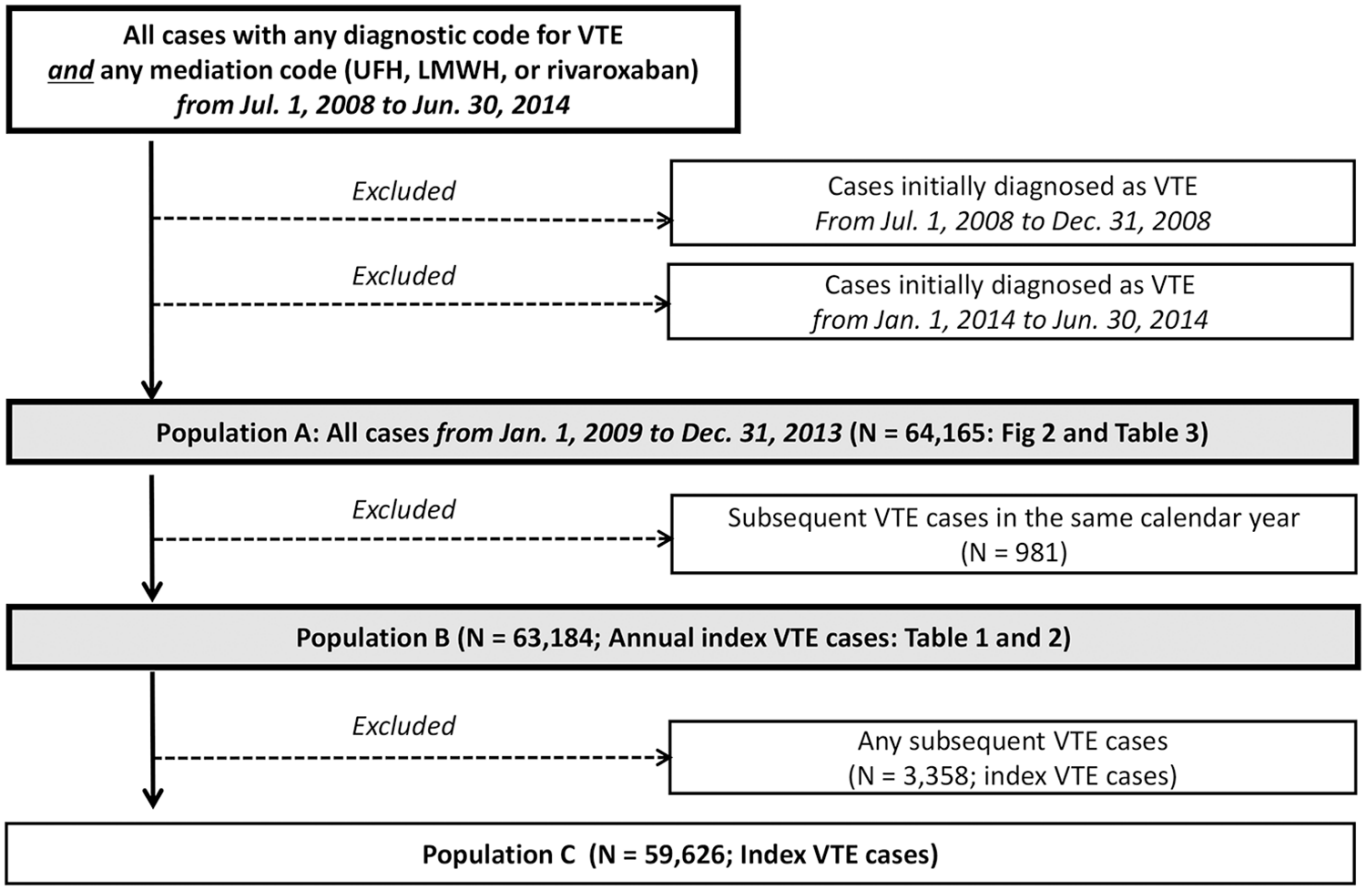
Overall flow of analyses.

First, inpatient and outpatient cases with both diagnostic and medication codes for VTE from July 1, 2008 to June 30, 2014 were extracted from the HIRA data. The date of VTE diagnosis was defined as the first day of simultaneous assignment of diagnostic and medication codes. Then, subjects whose date of diagnosis was before January 1, 2009 or between January 1, 2014 and June 30, 2014 were excluded (population A; Fig 1). Population A was used in the analysis of treatment pattern. To calculate respective annual incidence and relative risk of VTE according to calendar year, age, and gender, patients diagnosed with VTE who experienced a second event in the same calendar year were excluded (population B). Then, any subsequent VTE cases during the study period were excluded (Population C).

### Analyses of incidences of VTE and pattern of VTE treatment

Annual ASR and relative risk of VTE according to calendar year, age, and gender were analyzed in Population B. We did not perform this analysis in Population A to eliminate the possibility that some patients, for whom the initial anticoagulant treatment failed and who then received second-line anticoagulants, would be counted as two VTE cases. The annual ASR and relative risk of VTE incidence were also not calculated in Population C because in a previous study VTE had a cumulative risk of relapse of 12.9% and 22.8% at 1 year and 5 years, respectively [19], and thus medical and social impacts of VTE occurrence would not be limited to an individual’s first VTE diagnosis.

Analyses of the pattern of VTE treatments (*i*.*e*., medication or procedures) were performed for Population A. All medication codes for anticoagulants approved for VTE in Korea [UFH, LMWH, DOAC (*i*.*e*., rivaroxaban), and warfarin] prescribed within 6 months after VTE diagnosis were collected from HIRA databases. Patterns of anticoagulant use were analyzed and classified as the following: 1) UFH alone, 2) LMWH-based (LMWH alone, and UFH → LMWH), 3) warfarin-based (UFH and/or LMWH → warfarin), 4) rivaroxaban-based (rivaroxaban alone, UFH and/or LMWH → rivaroxaban), and 5) rivaroxaban and warfarin (prescription of both rivaroxaban and warfarin regardless of order prescribed with or without initial UFH or LMWH). Codes for procedures, including thrombectomy, thromboplasty, catheter-directed thrombolysis, and placement of inferior vena cava (IVC) filter, recorded from -1 to +6 months of the time of VTE diagnosis were searched and analyzed.

### Statistical analysis

Crude annual incidences (per 100,000 individuals) were determined using the number of individuals with DVT and/or PE (Population B) as numerator and the number of people in the Korean population for each year based on the HIRA database as the denominator. ASR of DVT and PE were directly adjusted to mid-year population in 2011. Confidence interval (CI) estimates were based on Poisson distribution. Differences in the incidence and CI by age, sex, and year were estimated by Poisson distribution. Average annual percent change in incidence was calculated as described previously [20]. *P* < 0.05 was considered statistically significant.

## Results

### Annual incidence of VTE

From January 2009 to December 2013, 64,165 incident cases (Population A), 63,184 annual index cases (Population B), and 59,626 index cases (Population C) of VTE were identified. Mean age- and sex-non-adjusted annual incidences of VTE, DVT, and PE during the entire study period were 23.4, 9.4, and 14.1 per 100,000 individuals, respectively. Age- and sex- adjusted annual incidences of VTE, DVT, and PE from 2009 to 2013 are shown in Table 1. The annual incidences increased steadily year by year. Increases were relatively steep between 2012 and 2013, especially in women. In 2009, incidences of VTE, DVT, and PE were 21.3, 8.1, and 13.2 per 100,000 individuals, respectively. In 2010, they increased to 22.6, 8.3, and 14.3 per 100,000 individuals, respectively. In 2011, the incidences increased to 24.2, 9.7, and 14.6 per 100,000, respectively. They further increased to 25.6, 10.3, and 15.3 per 100,000, respectively, in 2012 and to 29.2, 12.7, and 16.6 per 100,000, respectively, in 2013 (Table 1). Average annual percent changes in VTE, DVT, and PE during the study period were +7.85%, +11.80%, and +5.40%, respectively.

**Table 1:** Annual incidence of VTE, DVT, and PE in the Korean population from 2009 to 2013.

Year	Annual incidence (per 100,000 population)														
	2009			2010			2011			2012			2013		
	VTE	DVT	PE	VTE	DVT	PE	VTE	DVT	PE	VTE	DVT	PE	VTE	DVT	PE
**Age group**															
**Male**															
0–9	0.35	0.27	0.08	0.12	0.08	0.04	0.17	0.12	0.04	0.21	0.21	0.00	0.29	0.17	0.13
10–19	0.78	0.33	0.44	0.81	0.36	0.45	1.00	0.54	0.46	1.33	0.65	0.68	1.29	0.58	0.71
20–29	4.02	1.93	2.09	3.68	1.43	2.25	5.23	2.26	2.98	4.41	2.19	2.22	4.78	2.28	2.50
30–39	7.05	3.71	3.34	6.35	3.25	3.09	6.95	3.34	3.62	7.91	3.43	4.49	9.12	4.69	4.43
40–49	11.28	5.45	5.83	10.28	4.98	5.29	12.77	6.51	6.26	13.18	6.62	6.56	14.62	7.64	6.98
50–59	23.38	11.05	12.32	25.47	11.71	13.75	26.63	12.93	13.70	27.57	12.70	14.87	29.71	15.03	14.68
60–69	60.55	24.07	36.47	67.55	25.41	42.14	68.36	29.49	38.87	71.90	29.75	42.15	78.21	34.48	43.74
70–79	129.50	40.67	88.83	137.31	44.67	92.64	144.74	47.22	97.52	150.01	48.95	101.06	163.63	59.89	103.74
≥ 80	178.84	47.17	131.67	200.20	57.62	142.58	219.16	81.71	137.45	222.60	71.65	150.95	240.88	81.46	159.42
Subtotal*	19.91	7.81	12.10	21.00	8.06	12.94	22.57	9.38	13.19	23.43	9.38	14.05	25.57	11.08	14.49
**Female**															
0–9	0.04	0.04	0.00	0.39	0.22	0.17	0.27	0.09	0.18	0.31	0.27	0.04	0.22	0.13	0.09
10–19	0.59	0.31	0.28	0.71	0.53	0.19	0.76	0.44	0.32	0.56	0.26	0.29	0.44	0.27	0.17
20–29	2.69	1.27	1.42	2.71	1.55	1.16	2.86	1.49	1.37	2.78	1.36	1.42	3.58	2.03	1.56
30–39	4.43	1.97	2.46	5.20	2.48	2.72	5.77	3.09	2.68	7.60	4.35	3.24	7.76	3.93	3.83
40–49	9.22	4.99	4.23	9.51	4.86	4.65	10.92	5.79	5.12	12.15	6.52	5.62	13.50	6.93	6.57
50–59	18.30	8.43	9.87	19.76	8.88	10.88	21.16	9.75	11.41	22.73	11.02	11.71	25.45	12.79	12.66
60–69	60.10	22.98	37.12	57.82	20.41	37.41	63.44	24.55	38.89	64.94	26.81	38.13	79.71	36.52	43.20
70–79	127.00	42.00	85.01	136.13	41.87	94.26	139.42	46.45	92.97	149.98	53.14	96.84	183.89	72.61	111.29
≥ 80	144.43	41.74	102.68	165.88	46.50	119.38	184.31	61.95	122.36	194.20	62.81	131.39	230.69	87.63	143.07
Subtotal*	22.74	8.45	14.29	24.16	8.56	15.61	25.93	10.00	15.93	27.71	11.13	16.58	32.91	14.24	18.67
**Total**^†^	21.3	8.1	13.2	22.6	8.3	14.3	24.2	9.7	14.6	25.6	10.3	15.3	29.2	12.7	16.6

*Age adjusted to 2011 population.

^†^Age and sex adjusted to 2011 population.

VTE, venous thromboembolism; DVT, deep vein thrombosis; PE, pulmonary embolism

### Annual incidence of VTE by age and sex

Annual incidences of VTE, DVT, and PE increased with age (Table 2 and S1 Fig). When compared to the 30–39-year old population, the relative risk for VTE was 9.89 (95% CI: 9.50–10.29, *P* < 0.001) for those 60–69 years of age, 21.64 (95% CI: 20.81–22.51, *P* < 0.001) for those 70–79 years of age, and 28.57 (95% CI: 27.41–29.79, *P* < 0.001) for those 80–89 years of age (Table 2).

**Table 2:** Relative risk of VTE, DVT, and PE according to calendar year, age, and sex.

	VTE		DVT		PE	
	RR (95% CI)	*P*	RR (95% CI)	*P*	RR (95% CI)	*P*
**Age group (years)**						
0–9	0.03 (0.03–0.05)	<0.0001	0.05 (0.03–0.06)	<0.0001	0.02 (0.01–0.04)	<0.0001
10–19	0.12 (0.11–0.14)	<0.0001	0.13 (0.11–0.15)	<0.0001	0.12 (0.1–0.14)	<0.0001
20–29	0.54 (0.51–0.58)	<0.0001	0.52 (0.47–0.57)	<0.0001	0.57 (0.52–0.62)	<0.0001
30–39 (referent)	1.00		1.00		1.00	
40–49	1.73 (1.65–1.81)	<0.0001	1.76 (1.65–1.88)	<0.0001	1.69 (1.58–1.8)	<0.0001
50–59	3.55 (3.4–3.7)	<0.0001	3.37 (3.17–3.58)	<0.0001	3.73 (3.52–3.96)	<0.0001
60–69	9.89 (9.5–10.29)	<0.0001	8.04 (7.59–8.52)	<0.0001	11.75 (11.11–12.43)	<0.0001
70–79	21.64 (20.81–22.51)	<0.0001	14.79 (13.97–15.65)	<0.0001	28.56 (27.04–30.17)	<0.0001
≥ 80	28.57 (27.41–29.79)	<0.0001	18.63 (17.5–19.83)	<0.0001	38.61 (36.45–40.89)	<0.0001
**Year**						
2009 (referent)	1.00		1.00		1.00	
2010	1.09 (1.06–1.12)	<0.0001	1.05 (1.01–1.1)	0.322	1.12 (1.08–1.16)	<0.0001
2011	1.21 (1.18–1.24)	<0.0001	1.26 (1.21–1.32)	<0.0001	1.18 (1.14–1.22)	<0.0001
2012	1.33 (1.3–1.36)	<0.0001	1.38 (1.33–1.44)	<0.0001	1.3 (1.25–1.34)	<0.0001
2013	1.57 (1.53–1.61)	<0.0001	1.76 (1.69–1.83)	<0.0001	1.45 (1.4–1.49)	<0.0001
**Sex**						
Male (referent)	1.00		1.00		1.00	
Female	1.19 (1.17–1.21)	<0.0001	1.15 (1.12–1.18)	<0.0001	1.21 (1.19–1.24)	<0.0001

DVT, deep vein thrombosis; PE, pulmonary embolism; RR, relative risk; CI, confidence interval

Both DVT and PE showed consistent increases in annual incidence irrespective of sex. The annual incidence of DVT in females increased more significantly than in males during the study period (S2 Fig). Being female was a risk for VTE, DVT, and PE, with relative risk of 1.19 (95% CI: 1.17–1.21, *P* < 0.001), 1.15 (95% CI: 1.12–1.18, *P* < 0.001), and 1.21 (95% CI: 1.19–1.24, *P* < 0.001), respectively (Table 2).

### Patterns of treatment for VTE

**Fig 2:**
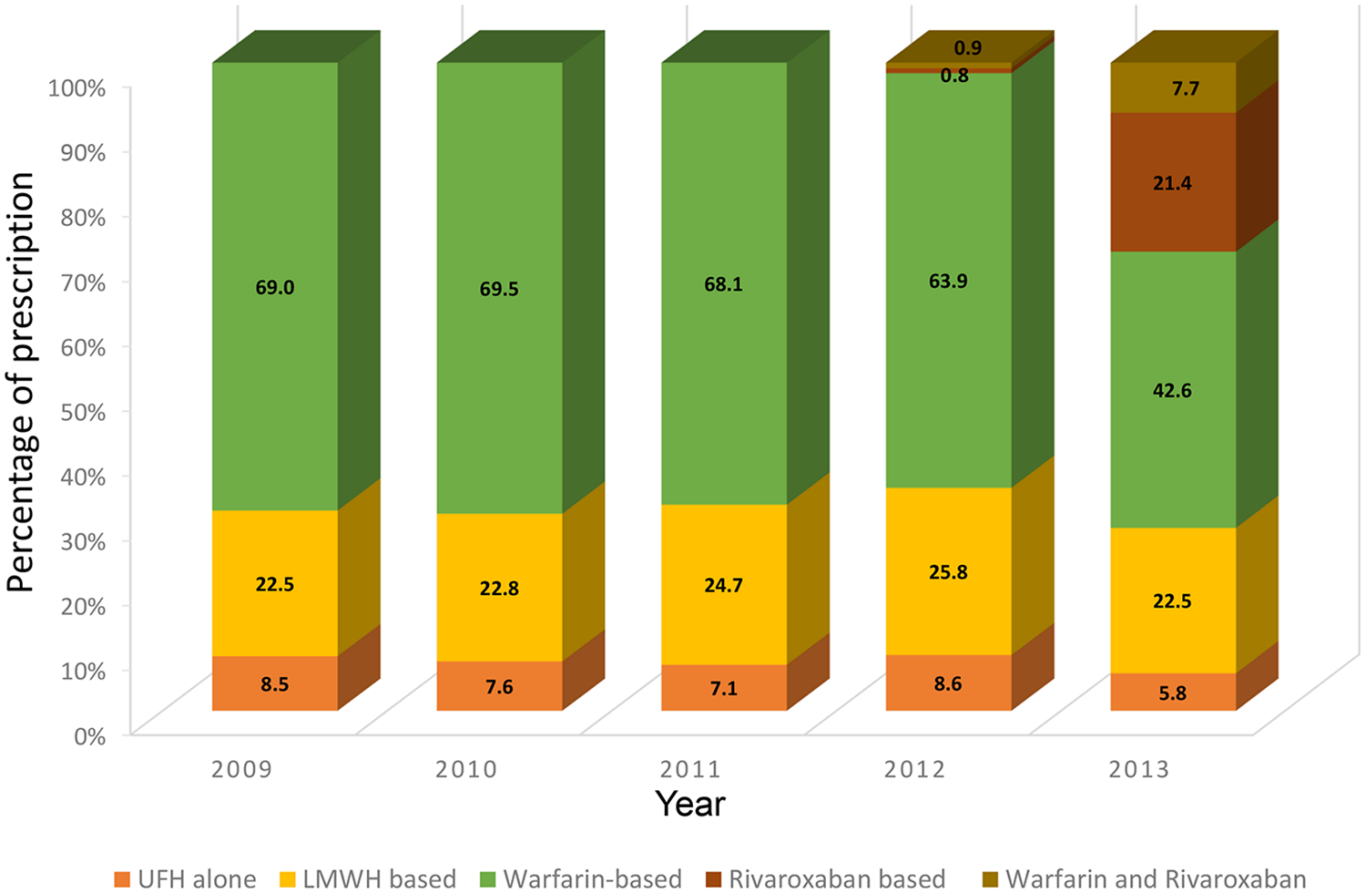
Pattern of prescription of anticoagulants during the study period.

Warfarin was the most frequently used anticoagulant therapy, followed by LMWH during the study period. Annual relative prescription rates of warfarin and LMWH decreased with the introduction of DOAC between 2012 and 2013 (warfarin, from 63.9% in 2012 to 42.6% in 2013, *P* < 0.001; LMWH, from 25.8% in 2012 to 22.5% in 2013, *P* < 0.001; Fig 2 and S1 Table). A relatively small number of subjects underwent procedures to treat VTE. Unlike the gradual increase in annual ASR *per se*, the proportion of subjects who underwent procedures decreased significantly (*P* < 0.001) during the study period. This was observed for thromboplasty, catheter-directed thrombolysis, intravenous thrombolysis, and IVC filter, but not for thrombectomy (*P* = 0.710, Table 3).

**Table 3:** Percentage of all VTE, DVT, and PE subjects who underwent procedures in each calendar year.

Year	% of subjects who received procedures among entire subjects diagnosed VTE in a year														
	2009			2010			2011			2012			2013		
	VTE	DVT	PE	VTE	DVT	PE	VTE	DVT	PE	VTE	DVT	PE	VTE	DVT	PE
Types of procedures done (between -1 and +6 months of diagnosis)*															
Thrombectomy	0.17	0.12	0.05	0.17	0.12	0.05	0.13	0.08	0.05	0.17	0.15	0.02	0.20	0.16	0.04
Thromboplasty	5.20	4.28	0.93	5.20	4.29	0.90	4.95	4.19	0.75	4.56	3.79	0.78	4.17	3.50	0.67
Thrombolysis (CD)	5.53	4.29	1.23	5.70	4.30	1.40	5.73	4.47	1.25	5.07	3.84	1.23	4.63	3.55	1.08
Thrombolysis (IV)	1.94	0.31	1.64	1.56	0.29	1.27	1.60	0.26	1.33	1.36	0.18	1.17	1.29	0.27	1.02
Inferior vena cava filter	8.63	4.67	3.96	8.85	4.42	4.42	8.48	4.71	3.77	8.16	4.53	3.63	6.76	3.79	2.97

*A patient might have received two or more procedures.

VTE, venous thromboembolism; DVT, deep vein thrombosis; PE, pulmonary embolism; CD, catheter-directed; IV, intravenous

## Discussion

A previous Korean population-based study reported that the ASR of DVT with PE per 100,000 individuals increased from 7.65 in 2004 to 12.32 in 2008 [16]. Based on those results and the results of the present study, the ASR in Korea has continuously increased since 2004. There was a steep increase in ASR of VTE between year 2008 in the previous study and 2009 (from 12.32 per 100,000 in 2008 to 23.0 per 100,000 in 2009). This may be due to the following reasons. First, this increase may be due to differences in the methods used to identify VTE subjects. In the present study, all subjects who had a diagnostic code of VTE (from the main to the fourth minor diagnostic code) were included even if it was not the main diagnostic code. This is because subjects who have VTE frequently have comorbidities; this is the case primarily in the elderly. It is likely that many subjects were newly diagnosed with VTE during hospitalization for other conditions, such as cancer, fracture, or cardiovascular problems. Therefore, we believe that our data is a more reliable indicator of the annual incidence of VTE in an Asian population. Second, some relapsed cases of VTE were included in the present study to estimate ASR if the relapse had not occurred in the same calendar year as the initial diagnosis. Relapse of VTE is not uncommon. Indeed, it is an important issue, with medical and economic impacts. Third, the results might be due to a true substantial increase in VTE incidence. Although there are differences between the two studies, it was obvious that the ASR of VTE in Korea had continuously increased over the past decade.

Several factors might have contributed to this increase. First, an increase in cancer and other morbidities might have had an impact on the increase in VTE because active cancer is one of the most common factors affecting the incidence of VTE [21]. Age- and sex-adjusted cancer incidence rate from all sites in Korea have undergone a gradual increase, from 210.5 per 100,000 in 1999 to 290.5 per 100,000 in 2013 [22]. In addition, the prolonged survival of cancer patients, rather than an increase in the incidence of cancer itself, might be the greater contributor to the increase in the ASR of VTE. However, the increase in the incidence and survival of patients with cancer cannot sufficiently explain the substantial increase in the ASR of VTE during the study period. It is possible that increased physician and public awareness of VTE may have led to significantly increased detection. Although such awareness cannot be quantified, VTE has now become a common and well-known disease in Korea, and more primary care practitioners have become familiar with its diagnosis and treatment. Intriguingly, there was a relatively steep increase in ASR in 2013, coinciding with the emergence of DOAC. Active marketing of a new treatment option and physician interest in DOAC might have contributed to the increased detection of VTE.

The results of this study provide an important perspective on the increasing incidence of VTE in countries with increasing socioeconomic capacity, mainly in Asia. The annual incidence of VTE in the general Asian population is still significantly lower than in Western populations, where the annual incidence of VTE consistently exceeds 100 per 100,000 individuals (Table 4), although the studies used heterogeneous definitions of VTE, DVT, and PE. However, the results of the present study suggest that the incidence of VTE in Asian populations will continue to increase in the future. It is difficult to predict the extent to which VTE incidence will increase in Korea or other Asian countries under similar situations. Further, there are clear genetic and racial differences between Western and Asian populations, particularly in terms of inherited thrombophilia [23]. Nevertheless, it is clearly necessary to take measures against a significant increase of VTE in Asian countries.

**Table 4:** Reported VTE incidence in studies conducted in the past decade.

					Incidence (per 100,000) *		
	Location	Study Period	Study Design	Size of Population	VTE	DVT	PE
**Western Studies**							
Delluc et al (2016) [3]	Western France	Mar 2013–Feb 2014	Prospective cohort	among 367,911 inhabitants	157	119.8	64.2
Bouée et al (2016) [4]	France	2 years (2010–2011)	Population-based	around 600,000 individuals	184		
Alotaibi et al (2016) [5]	Alberta (Canada)	Apr 2002 –Mar 2012	Population-based	over 4 million in 2014	138	100	38
Johansson et al (2014) [6]	Västerbotten (Sweden)	2006	Population-based	204,836 residents	167	76.6	78.6
Tagalakis et al (2013) [1]	Québec (Canada)	2000–2009	Population-based	over 74 million person-years	122	78	45
Spencer et al (2009) [7]	Worcester, MA (USA)	1999, 2001, and 2003	Population-based	477,800 residents in 2000	114	95	34
Heit et al (2017) [24]	Olmsted, MN (USA)	1981–2010	Population-based	493,000 individuals	123	47	62
**Asian Countries**							
Present study	Korea	2009–2013	Population-based	Around 50 million/year	23.4	9.4	14.1
Jang et al (2011) [16]	Korea (our previous)	2004–2008	Population-based	around 49 million/year	13.8	5.31	7.01
Sakuma et al (2009) [25]	Japan	Aug–Sep 2006	Questionnaire	6,135 replies		11.6	6.19
Molina et al (2011) [9]	Singapore	2006	Cohort (3 major hospital)	98,121 admitted patients	57		15
Lee et al (2011) [26]	Taiwan	2002	Population-based	around 22.5 million/year	16.5		4.8
Cheuk et al (2004) [27]	Hong Kong	2000–2001	Retrospective	2,082,245 admissions	21	17.1	3.9

*In some studies, the sum of DVT and PE is not equal to the number of VTE because heterogeneous definitions of VTE, DVT, and PE were used.

VTE, venous thromboembolism; DVT, deep vein thrombosis; PE, pulmonary embolism

Based on the results of global studies comparing rivaroxaban to enoxaparin/vitamin K antagonist therapy for VTE [28, 29], rivaroxaban has been covered by the Korean NHI service since January 1, 2013. As expected, prescription of a DOAC has significantly increased, whereas warfarin prescriptions have declined accordingly. This might be because most of the population would benefit from the lower cost of a DOAC reimbursed by NHI service for 6 months of treatment, in addition to the ease of use, safety, and reduced length of hospital stay with the use of DOAC [30, 31].

The present study has some limitations. First, 5.8–8.6% of subjects with a diagnostic code of VTE were prescribed only UFH each year. Some of these might be serious or fatal cases. For example, some subjects died soon after the diagnosis of VTE, whereas some subjects discontinued anticoagulation therapy early due to major bleeding. However, there is a lack of data regarding survival of these subjects. Therefore, the exact reason for using UFH only was unknown. Second, the definition of relapse used in the present study was not based on the results of imaging. A widely accepted definition of VTE recurrence is "VTE of a site that was either previously uninvolved or had interval documentation of incident VTE resolution", proposed by Heit *et al*. [32]. In the present study, the results of imaging studies of the location or size change of a thrombus could not be retrieved. Third, data for long-term anticoagulation in patients with a high risk of relapse, such as those with cancer and antiphospholipid syndrome, could not be analyzed. Beyond 6 months after the initial diagnosis of VTE, only the cost of warfarin can be reimbursed in Korea. Patients who are treated with LMWH or rivaroxaban for indefinite anticoagulation after the first 6 months must pay the cost by themselves. Therefore, their course and outcome could not be analyzed using the HIRA database. Fourth, although the HIRA database included both hospitalized and outpatient VTE patients, it was not feasible to clearly distinguish the two types of VTE. Finally, because we defined VTE as the association of a code for VTE and a code for anticoagulant, a minority of patients with VTE but left untreated, such as patients with asymptomatic distal DVT, or those for whom anticoagulant therapy was contraindicated, could be under-recognized.

In conclusion, ASR of VTE tended to increase from 2009 to 2013, reflecting improved survival of patients with cancer and other morbidities, and most importantly, increased awareness and detection of VTE. Introduction and good accessibility to DOAC resulted in significant changes in the pattern of anticoagulant prescription.

## Supporting information

S1 Fig Annual incidence of VTE, DVT, and PE according to age group. (TIF)

S2 Fig Annual incidence of VTE, DVT, and PE by sex. (**A**) Annual incidence of VTE by sex. (**B**) Annual incidence of DVT by sex. (**C**) Annual incidence of PE by sex.
(TIF)

S1 Table Proportion of anticoagulant therapy over the study period (2009–2013). (PDF)
