# Supplementary figures and images for: Incidence of venous thromboembolism in Korea from 2009 to 2013
Source: PLoS One. 2018 Jan 25;13(1):e0191897. doi: 10.1371/journal.pone.0191897 (PMC5785001; doi:10.1371/journal.pone.0191897)

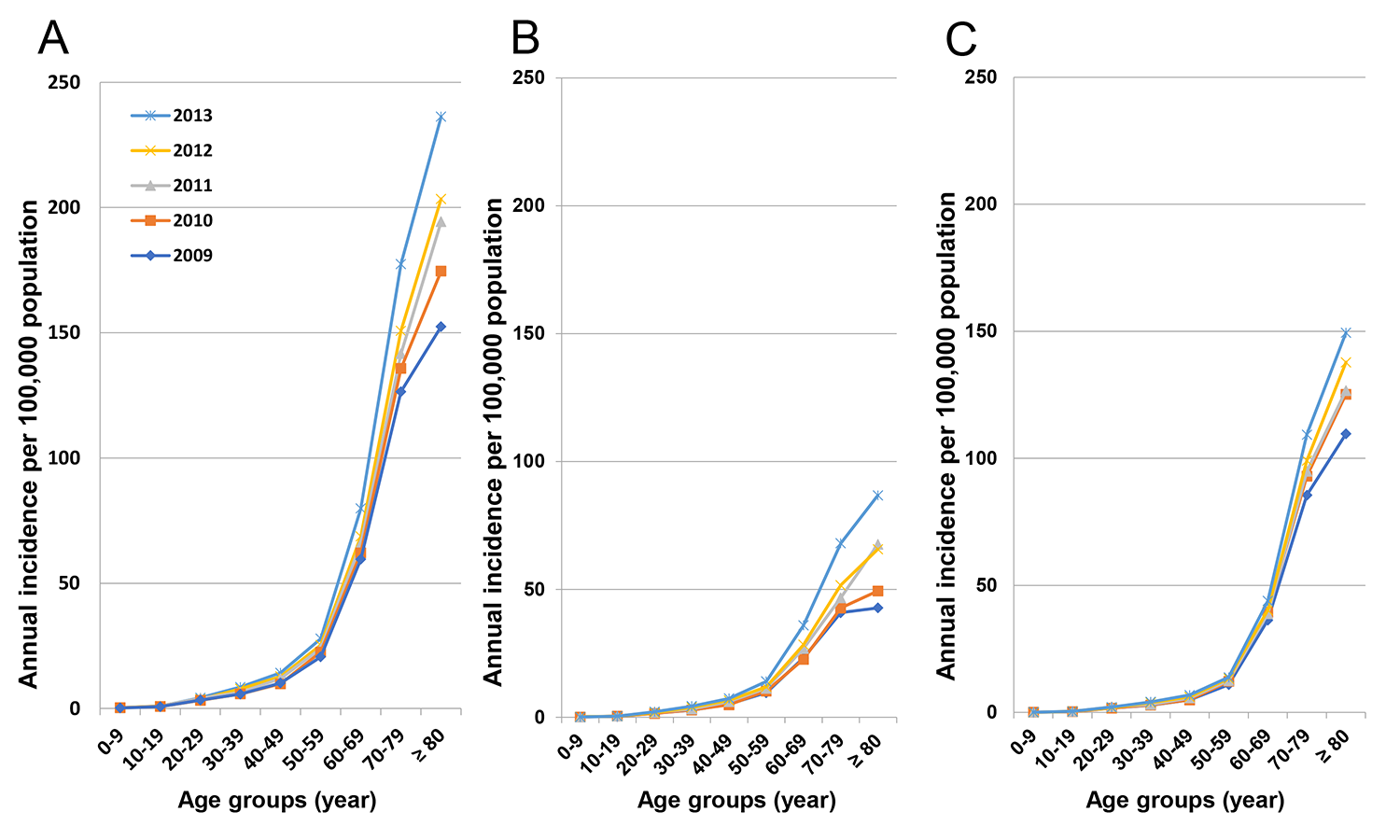

Supplement: S1 Fig — (TIF) [file pone.0191897.s001.tif]

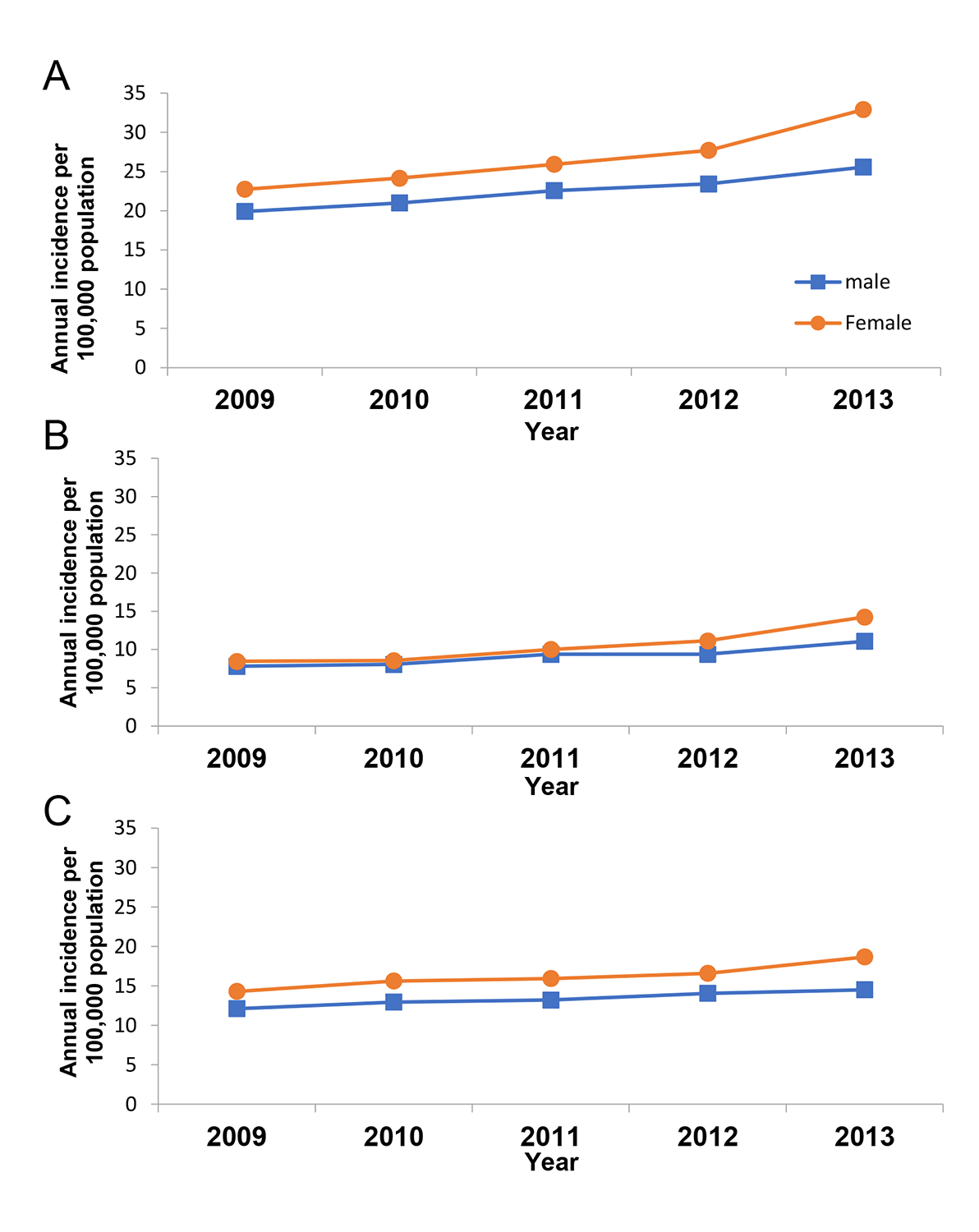

Supplement: S2 Fig — (A) Annual incidence of VTE by sex. (B) Annual incidence of DVT by sex. (C) Annual incidence of PE by sex. (TIF) [file pone.0191897.s002.tif]
