# Supplementary material for: Incidence of venous thromboembolism in Korea from 2009 to 2013
Source: PLoS One. 2018 Jan 25;13(1):e0191897. doi: 10.1371/journal.pone.0191897 (PMC5785001; doi:10.1371/journal.pone.0191897)
Supplement: S1 Table — (PDF) [file pone.0191897.s003.pdf]

S1 Table. Proportion of anticoagulative treatment over the study period (2009 to 2013)

	%														
Year	2009			2010			2011			2012			2013		
	VTE	DVT	PE	VTE	DVT	PE	VTE	DVT	PE	VTE	DVT	PE	VTE	DVT	PE
Types of anticoagulation used (within 3 months after the time of diagnosis)															
UFH alone	8.5	7.3	9.2	7.6	7.7	7.6	7.1	6.4	7.7	8.6	5.3	10.9	5.8	4.0	7.2
LMWH-based															
LMWH alone	15.5	18.7	13.5	15.9	18.9	14.1	17.2	21.3	14.4	18.9	25.9	14.2	16.2	18.9	14.2
UFH→LMWH	7.0	7.8	6.5	6.9	8.0	6.3	7.6	9.6	6.2	7.0	7.2	6.8	6.2	5.8	6.6
Warfarin-based															
UFH→Warfarin	19.5	18.6	20.1	17.5	16.3	18.2	16.9	15.6	17.8	14.5	13.5	15.2	11.2	8.7	13.2
LMWH→Warfarin	31.0	30.6	31.2	32.1	29.8	33.4	31.4	28.8	33.2	31.3	29.9	32.2	20.2	15.6	23.7
UFH and (↔) LMWH→Warfarin	18.5	17.1	19.5	19.9	19.4	20.3	19.8	18.4	20.7	18.1	16.9	18.9	11.1	8.2	13.4
Rivaroxaban based															
Rivaroxaban alone	0.0	0.0	0.0	0.0	0.0	0.0	0.0	0.0	0.0	0.6	0.0	1.0	12.3	20.6	5.9
UFH→Rivaroxaban	0.0	0.0	0.0	0.0	0.0	0.0	0.0	0.0	0.0	0.0	0.1	0.0	2.9	3.7	2.2
LMWH→Rivaroxaban	0.0	0.0	0.0	0.0	0.0	0.0	0.0	0.0	0.0	0.1	0.1	0.1	4.0	4.3	3.8
UFH↔LMWH→Rivaroxaban	0.0	0.0	0.0	0.0	0.0	0.0	0.0	0.0	0.0	0.0	0.1	0.0	2.3	2.5	2.2
Rivaroxaban and warfarin															
Rivaroxaban↔Warfarin	0.0	0.0	0.0	0.0	0.0	0.0	0.0	0.0	0.0	0.0	0.0	0.0	1.0	1.5	0.7
UFH→Rivaroxaban↔Warfarin	0.0	0.0	0.0	0.0	0.0	0.0	0.0	0.0	0.0	0.2	0.3	0.1	1.5	1.5	1.5
LMWH→Rivaroxaban↔Warfarin	0.0	0.0	0.0	0.0	0.0	0.0	0.0	0.0	0.0	0.4	0.5	0.3	2.8	2.7	3.0
UFH↔LMWH→Rivaroxaban↔Warfarin	0.0	0.0	0.0	0.0	0.0	0.0	0.0	0.0	0.0	0.3	0.3	0.3	2.4	2.2	2.5
Total (%)	100	100	100	100	100	100	100	100	100	100	100	100	100	100	100

VTE, venous thromboembolism; DVT, deep vein thrombosis; PE, pulmonary embolism; UFH, unfractionated heparin; LMWH, low-molecular-weight heparin
